# WHIGET and TETRAS Ratings of Action Tremor in Patients with Essential Tremor: Substantial Association and Agreement

**DOI:** 10.5334/tohm.874

**Published:** 2024-03-27

**Authors:** Adreanna B. Hernandez, Diane S. Berry, Natalie Grill, Talía M. Hall, Allison Burkes, Ali Ghanem, Vibhash D. Sharma, Elan D. Louis

**Affiliations:** 1 Medical School, University of Texas Southwestern Medical Center, Dallas, Texas, USA; 2 Department of Neurology, University of Texas Southwestern Medical Center, Dallas, Texas, USA; 3 Peter O’Donnell Jr. Brain Institute, University of Texas Southwestern Medical Center, Dallas, Texas, USA

**Keywords:** essential tremor, tremor ratings, WHIGET, TETRAS, reliability, tremor severity

## Abstract

**Background:**

Evaluating tremor severity is a critical component of diagnosing and clinically managing patients with essential tremor (ET). We examined the comparability of tremor severity ratings derived from two frequently used tremor rating scales: the Washington Heights-Inwood Genetic Study of Essential Tremor (WHIGET) rating scale and the Tremor Research Group Essential Tremor Rating Scale (TETRAS).

**Methods:**

A trained assistant administered and videotaped a neurological examination, including eight items assessing upper limb action tremor (arms outstretched, arms in the wingbeat position, finger-nose-finger maneuver, and drawing of Archimedes spirals). An experienced movement disorders neurologist reviewed the videos and assigned WHIGET and TETRAS ratings. We calculated associations between TETRAS and WHIGET ratings using Spearman rank order correlations. Subsequently, we collapsed these ratings into four tremor severity categories (absent, mild, moderate, severe) and then two broader tremor severity categories (absent/mild, moderate/severe). We calculated weighted Kappa coefficients to assess agreement between category assignments based on the TETRAS and the WHIGET.

**Results:**

Spearman’s *r*’ s were significant for all items (*p’s* ≤ 0.001, mean *r* = 0.89). Weighted Kappa’s revealed substantial to near perfect agreement for all eight items (mean *k* = 0.86, range = 0.64 to 1.00).

**Conclusion:**

Analyses revealed substantial strength of association and substantial to near perfect agreement between items rated with the WHIGET and TETRAS scales. These data indicated that ratings provided by each scale are highly comparable.

## Introduction

Essential tremor (ET) is a chronic, slowly progressive neurologic disease whose primary feature is kinetic tremor involving the hands and arms [1,2,3]. This tremor is typically mildly asymmetric [4] and is often associated with a postural and/or intentional component [5,6]. Tremor generally worsens in severity with time, with an additional long-term tendency to spread from isolated upper limb involvement to involvement of cranial structures in many patients [7,8,9,10].

Assessment of the presence and severity of tremor is a critical component of the initial diagnosis and clinical management of patients with ET [11]. The Washington Heights-Inwood Genetic Study of Essential Tremor (WHIGET) rating scale was originally designed for the purpose of defining a tremor severity threshold for confidently diagnosing ET vs. physiologic tremor in population-based studies [12,13]. The scale is both reliable and valid [14,15]. The scale includes ratings of both postural and kinetic tremors of the upper extremities. Since its initial publication, the WHIGET scale has been modified and enhanced (e.g., increasing the range of possible scores). The rating scale, and associated diagnostic criteria, have been used and continue to be used in numerous research studies [16,17,18,19,20,21,22,23,24,25,26].

The Tremor Research Group Essential Tremor Assessment Scale (TETRAS) is a more recently developed and validated scale designed for the clinical assessment of individuals with ET [27]. It is being used increasingly in tremor research [28,29,30,31].

To our knowledge, there has been no direct comparison of the ratings derived through the use of these two scales. As a result, it is not clear to what extent ratings from one scale would reflect those from the other, making comparisons across studies that use only one of these more difficult. The goal of the present analyses is to assess the agreement between WHIGET and TETRAS ratings of kinetic and postural tremor in the upper limbs. Specifically, we (1) evaluated tremor in ET cases using each scale (2) calculated the level of association between the numerical scores assigned to these cases by each scale, and (3) calculated the agreement between the conceptual levels of tremor severity (i.e., mild, moderate, severe) assigned by each scale. Our overarching goal was to enhance comparisons between studies that use one scale or the other.

## Methods

### Overview

Participants were enrolled in an ongoing prospective, longitudinal study of cognitive performance (Clinical Pathological Study of Cognitive Impairment in Essential Tremor [COGNET]; National Institutes of Health Award #R01 NS086736). Eligibility requirements were (1) a diagnosis of ET; (2) a baseline age of at least 55 years; (3) no history of brain surgery as treatment for ET; and (4) an agreement to become a future brain donor. Cases took part in six evaluations: baseline, and baseline plus 18, 36, 54, 72, and 90 months. The study was approved by the Yale University, Columbia University, and University of Texas Southwestern Medical Center Institutional Review Boards. All cases provided written, informed consent.

A trained research assistant administered the evaluations during home visits. Each visit involved the completion of demographic and clinical questionnaires and a videotaped neurological examination that included detailed assessments of tremor, as documented previously [32,33].

All cases received a clinical diagnoses of ET assigned by an experienced movement disorders neurologist using WHIGET criteria, which are both reliable [14] and valid [15]. All cases also fulfilled Consensus criteria for ET, which are less rigorous in the sense that they do not specify a minimum tremor severity for ET [34].

For these analyses, our sample comprised 80 ET cases who were enrolled in the COGNET study between September, 2021 (the launching of this reliability analysis) and July, 2023 (successful enrollment of 80 ET cases for this analysis). One case was excluded due to incomplete data, leaving a sample of 79 cases for these analyses.

#### Demographic and Clinical Questionnaires

During each evaluation, questionnaires were administered to obtain information about basic demographics (i.e., age, sex, race, years of education) as well as details of tremor history. The latter included age of tremor onset, and tremor duration (current age minus age of tremor onset).

#### Videotaped Neurological Examination

Among other items, the videotaped neurological examination included the following maneuvers in each arm: sustained posture (first with arms outstretched and then in the wingbeat position), finger-nose-finger maneuver, and drawing of Archimedes spirals [12].

#### Assignment of TETRAS and WHIGET Ratings

An experienced movement disorders neurologist viewed the videotaped neurological examination and assigned ratings of eight items using the WHIGET and TETRAS rating scales (bilateral assessments of postural tremor with arms outstretched, postural tremor during the wingbeat position, kinetic tremor during the finger-nose-finger maneuver, and kinetic tremor during the drawing of Archimedes spirals) (Table 1).

**Table 1:** WHIGET and TETRAS Rating Scales: Items and Scale Values.


EIGHT ITEMS	SCALE VALUES (WHIGET)	SCALE VALUES (TETRAS)

Spiral, dominant	0, 0.5, 1, 1.5, 2, 3, 4	0, 1, 2, 3, 4

Spiral, non-dominant	0, 0.5, 1, 1.5, 2, 3, 4	0, 1, 2, 3, 4

Finger to nose, dominant	0, 0.5, 1, 1.5, 2, 3, 4	0, 1, 1.5, 2, 2.5, 3, 3.5, 4

Finger to nose, non-dominant	0, 0.5, 1, 1.5, 2, 3, 4	0, 1, 1.5, 2, 2.5, 3, 3.5, 4

Outstretched, dominant	0, 0.5, 1, 1.5, 2, 3	0, 1, 1.5, 2, 2.5, 3, 3.5

Outstretched, non-dominant	0, 0.5, 1, 1.5, 2, 3	0, 1, 1.5, 2, 2.5, 3, 3.5

Wingbeat, dominant	0, 0.5, 1, 1.5, 2, 3	0, 1, 1.5, 2, 2.5, 3, 3.5

Wingbeat, non-dominant	0, 0.5, 1, 1.5, 2, 3	0, 1, 1.5, 2, 2.5, 3, 3.5


#### Statistical Analyses

For each specific tremor item, we initially examined the strength of the association between the numerical score yielded for each case by the WHIGET and TETRAS by calculating Spearman rank order correlations. Although these analyses assess similarity in the relative magnitude of numerical scores provided to cases by the WHIGET and TETRAS, they do not indicate more broadly whether the WHIGET and TETRAS classify tremor severity similarly (e.g., none, mild, moderate, severe). To assess this, an experienced movement disorders neurologist read the verbal description accompanying the numerical score for each TETRAS item (Table 2a–2c) and assigned the score to one of four categories: (1) no tremor, (2) mild tremor (3) moderate tremor, and (4) severe tremor. A parallel procedure assigned every WHIGET numerical score to the same four categories (Table 2). We then calculated weighted Kappa coefficients [35] to assess the level of agreement between the four category classification of TETRAS numerical scores and the parallel four category classification of WHIGET numerical scores.

**Table 2:** Original TETRAS and WHIGET Numerical Scale Values, and Corresponding Four and Two Level Tremor Severity Categories.


ORIGINAL TETRAS SCALE VALUE	CORRESPONDING FOUR LEVEL TETRAS TREMOR SEVERITY CATEGORY	CORRESPONDING TWO LEVEL TETRAS TREMOR SEVERITY CATEGORY	ORIGINAL WHIGET SCALE VALUE	CORRESPONDING FOUR LEVEL WHIGET TREMOR SEVERITY CATEGORY	CORRESPONDING TWO LEVEL WHIGET TREMOR SEVERITY CATEGORY

2A. POSTURAL TREMOR					

0.0 No tremor	1 (none)	1 (none/mild)	0.0 Absolutely no visible tremor	1 (none)	1 (none/mild)

1.0 Tremor is barely visible	2 (mild)	1 (none/mild)	0.5 Very low amplitude and almost never present	2 (mild)	1 (none/mild)

1.5 Tremor is visible, but <1 cm amplitude	2 (mild)	1 (none/mild)	1.0 Low amplitude tremor OR intermittent tremor	2 (mild)	1 (none/mild)

2.0 Tremor is 1–<3 cm amplitude	3 (moderate)	2 (moderate/severe)	1.5 Moderate amplitude AND clearly oscillatory, but only sometimes of moderate amplitude	3 (moderate)	2 (moderate/severe)

2.5 Tremor is 3–<5 cm amplitude	4 (severe)	2 (moderate/severe)	2.0 Moderate amplitude [1–2 cm] AND clearly oscillatory AND usually of moderate amplitude	3 (moderate)	2 (moderate/severe)

3.0 Tremor is 5–<10 cm amplitude	4 (severe)	2 (moderate/severe)	3.0 Large amplitude	4 (severe)	2 (moderate/severe)

3.5 Tremor is 10–<20 cm amplitude	4 (severe)	2 (moderate/severe)			

4.0 Tremor is >20 cm amplitude	4 (severe)	2 (moderate/severe)			

**2B. KINETIC TREMOR – FINGER-NOSE-FINGER MANEUVER**					

0.0 No tremor	1 (none)	1 (none/mild)	0.0 Absolutely no visible tremor	1 (none)	1 (none/mild)

1.0 Tremor barely visible	2 (mild)	1 (none/mild)	0.5 Very low amplitude and almost never present	2 (mild)	1 (none/mild)

1.5 Tremor visible, but <1 cm amplitude	2 (mild)	1 (none/mild)	1.0 Low amplitude tremor OR intermittent tremor	2 (mild)	1 (none/mild)

2.0 Tremor is 1–<3 cm amplitude	3 (moderate)	2 (moderate/severe)	1.5 Moderate amplitude AND clearly oscillatory, but only sometimes of moderate amplitude	3 (moderate)	2 (moderate/severe)

2.5 Tremor is 3–<5 cm amplitude	4 (severe)	2 (moderate/severe)	2.0 Moderate amplitude [1–2 cm] AND clearly oscillatory AND usually of moderate amplitude	3 (moderate)	2 (moderate/severe)

3.0 Tremor is 5–<10 cm amplitude	4 (severe)	2 (moderate/severe)	3.0 Large amplitude	4 (severe)	2 (moderate/severe)

3.5 Tremor is 10–<20 cm amplitude	4 (severe)	2 (moderate/severe)	4.0 Extremely large tremor	4 (severe)	2 (moderate/severe)

4.0 Tremor is >20 cm amplitude	4 (severe)	2 (moderate/severe)			

**2C. KINETIC TREMOR – ARCHIMEDES SPIRAL**					

0 Normal	1 (none)	1 (none/mild)	0.0 Absolutely no visible tremor	1 (none)	1 (none/mild)

1 Slight; tremor barely visible	2 (mild)	1 (none/mild)	0.5 Very low amplitude and almost never present	2 (mild)	1 (none/mild)

2 Mild; obvious tremor	3 (moderate)	2 (moderate/severe)	1.0 Low amplitude tremor OR intermittent tremor	2 (mild)	1 (none/mild)

3 Moderate; portions of figure not recognizable	4 (severe)	2 (moderate/severe)	1.5 Moderate amplitude [1–2 cm] AND clearly oscillatory but only sometimes of moderate amplitude	3 (moderate)	2 (moderate/severe)

4 Severe; figure not recognizable	4 (severe)	2 (moderate/severe)	2.0 Moderate amplitude AND clearly oscillatory and usually of moderate amplitude	3 (moderate)	2 (moderate/severe)

			3.0 Large amplitude	4 (severe)	2 (moderate/severe)

			4.0 Extremely large amplitude	4 (severe)	2 (moderate/severe)


This four-level classification was subsequently collapsed into a two-level classification. To accomplish this, TETRAS and WHIGET cases were re-categorized as displaying either (1) no or mild tremor versus (2) moderate or severe tremor (Table 2a–2c). We then calculated a new set of weighted Kappa coefficients that reflected agreement between cases’ assignment to these two categories.

## Results

The sample distributions of demographic and clinical characteristics and individual TETRAS and WHIGET items are shown in Table 3a and 3b.

**Table 3:** Characteristics of Cases.


3A. DEMOGRAPHIC AND CLINICAL FEATURES^a^								

Age (years)	81.6 ± 7.2							

Sex (female)	50 (63.3)							

Education (years)	16.2 ± 2.5							

Race (Caucasian)	76 (100.0)							

Age of tremor onset (years)	41.3 ± 21.5							

Tremor duration^b^ (years)	40.3 ± 20.4							

**3B. WHIGET AND TETRAS RATINGS**								

**ITEMS**	**WHIGET RATINGS**				**TETRAS RATINGS**			
	
	**OBSERVED MINIMUM**	**OBSERVED MAXIMUM**	**MEAN**	**MEDIAN**	**OBSERVED MINIMUM**	**OBSERVED MAXIMUM**	**MEAN**	**MEDIAN**

Spiral, dominant	0.0	4.0	1.8	2.0	0.0	4.0	2.0	2.0

Spiral, non-dominant	0.0	4.0	1.9	2.0	0.0	4.0	2.1	2.0

Finger to nose, dominant	0.0	3.0	1.5	1.5	0.0	2.5	1.6	2.0

Finger to nose, non-dominant	0.0	3.0	1.7	2.0	0.0	3.5	1.8	2.0

Outstretched, dominant	0.0	3.0	1.3	1.0	0.0	3.5	1.4	1.5

Outstretched, non-dominant	0.0	3.0	1.3	1.0	0.0	4.0	1.6	1.5

Wingbeat, dominant	0.0	3.0	1.5	1.5	0.0	4.0	1.7	2.0

Wingbeat, non-dominant	0.0	3.0	1.4	1.5	0.0	4.0	1.6	2.0


*Note*: Sample N = 79; number of observations may differ slightly among items due to occasional missing data. ^a^Values = Mean ± standard deviation or n (percentage). ^b^Age at time of tremor assessment – age of tremor onset.

Spearman rank order correlations (Table 4) revealed strong associations between the numerical scores yielded by the TETRAS and WHIGET for all items (*r*’s all ≥0.80 and ≤0.96, all *p*’s <0.001). In the assignment of the four level tremor severity categories based on the TETRAS and the WHIGET, the weighted Kappa coefficients (Table 4) revealed substantial to near perfect agreement (defined as a Kappa coefficient of >0.60 [36]) for all eight items (Kappa‘s = 0.76 to 0.99). Substantial to near perfect agreement in the assignment of the two level severity categories was again revealed for all items (Kappa’s = 0.64 to 1.00; Table 4).

**Table 4:** Spearman Rank Order Correlations and Weighted Kappa Coefficients: Association and Agreement Between TETRAS and WHIGET Assessments.


ITEMS	SPEARMAN’S RANK ORDER CORRELATION^a^	WEIGHTED KAPPA^b^	

		FOUR LEVEL^c^	TWO LEVEL^d^

Spiral, dominant	0.89***	0.83	0.86

Spiral, non-dominant	0.85***	0.76	0.64

Finger to nose, dominant	0.78***	0.78	0.77

Finger to nose, non-dominant	0.80***	0.76	0.78

Outstretched, dominant	0.96***	0.99	0.98

Outstretched, non-dominant	0.93***	0.90	0.90

Wingbeat, dominant	0.95***	0.96	1.00

Wingbeat, non-dominant	0.93***	0.95	0.92


*Note*: N = 79; *** *p* ≤ 0.001. ^a^Rank order correlations represent associations between TETRAS and WHIGET numerical scores assigned to cases. ^b^Kappa coefficient values of <0.00 are interpreted as poor agreement, of 0.00 to 0.20 as slight agreement, of 0.21 to 0.40 as fair agreement, of 0.41 to 0.60 as moderate agreement, of 0.61 to 0.80 as substantial agreement, and of 0.81 to 1.0 as near perfect agreement [36]. ^c^The four level weighted Kappa coefficients represent agreement between cases’ assignment to four tremor severity categories (no tremor, mild tremor, moderate tremor, severe tremor) based on TETRAS numerical scores, and cases’ assignment to those same four tremor severity categories based on WHIGET numerical scores. ^d^The two level weighted Kappa coefficients represent agreement between cases assignment to two tremor severity categories (no tremor/mild tremor versus moderate tremor/severe tremor) based on TETRAS numerical scores, and cases’ assignment to those same two tremor severity categories based on WHIGET numerical scores.

Granular data are provided in Supplemental Table 1.

## Discussion

Our analyses revealed substantial strength of association and substantial to near perfect agreement for items rated with the WHIGET and TETRAS scales. These data indicated that ratings provided by each scale are highly comparable. These results suggest that subtle differences in the development of the two scales do not substantially change the overall clinical assessment of patient tremor. Clinical and research implications of these findings are that the use of either of these scales is likely to yield similar results in clinical and research settings.

We acknowledge certain limitations to the present study. First, our study involved a single rater. Future studies may wish to utilize additional raters. Research that employs multiple raters, while more difficult to perform than that involving a single rater, provides a greater degree of methodological rigor, thus lending a greater degree of confidence in one’s findings. Second, while key features were compared across the two scales (several measure of both postural and kinetic tremor), WHIGET includes some maneuvers not assessed in TETRAS (e.g., pouring water, drinking water) and the converse is also true (e.g., dot approximation, sentence writing). Third, historically, the Fahn-Tolosa scale [37] has been a commonly used clinical severity rating scale in ET research. However, in this study, we chose to compare the WHIGET to the TETRAS because the use of the latter has become more widespread in recent years. Finally, tremor was rated using a videotape rather than live. However, video assessments offer a number of advantages over live assessments, with a major one being the ability to replay segments to assess subtle tremor phenomenology.

In summary, despite differences between the WHIGET and TETRAS, we demonstrate here that ratings of postural tremor (arms outstretched and in wingbeat position) and kinetic tremor (finger-nose-finger maneuver and while drawing spirals) are highly similar. Both scales assess tremor amplitude; the WHIGET also assesses the constancy of the tremor during the assessment window. Each provides valuable and comparable data in the evaluation of tremor severity among patients with ET.

## Additional File

The additional file for this article can be found as follows:

10.5334/tohm.874.s1 Supplemental Table 1. Cross-Tabulations of WHIGET and TETRAS Ratings.

## References

[1] Louis ED. Tremor. Contin. Minneap. *Minn*. 2019; 25: 959–975. DOI: 10.1212/CON.0000000000000748 31356289

[2] Brennan KC, Jurewicz EC, Ford B, Pullman SL, Louis ED. Is essential tremor predominantly a kinetic or a postural tremor? A clinical and electrophysiological study. Mov. Disord. Off. J. Mov. Disord. Soc. 2002; 17: 313–316. DOI: 10.1002/mds.10003 11921117

[3] Louis ED. The primary type of tremor in essential tremor is kinetic rather than postural: cross-sectional observation of tremor phenomenology in 369 cases. Eur. J. Neurol. 2013; 20: 725–727. DOI: 10.1111/j.1468-1331.2012.03855.x 22925197 PMC3511652

[4] Louis ED, Wendt KJ, Pullman SL, Ford B. Is essential tremor symmetric? Observational data from a community-based study of essential tremor. Arch. Neurol. 1998; 55: 1553–1559. DOI: 10.1001/archneur.55.12.1553 9865800

[5] Sternberg EJ, Alcalay RN, Levy OA, Louis ED. Postural and Intention Tremors: A Detailed Clinical Study of Essential Tremor vs. Parkinson’s Disease. Front. Neurol. 2013; 4: 51. DOI: 10.3389/fneur.2013.00051 23717300 PMC3650675

[6] Louis ED, Frucht SJ, Rios E. Intention tremor in essential tremor: Prevalence and association with disease duration. Mov. Disord. Off. J. Mov. Disord. Soc. 2009; 24: 626–627. DOI: 10.1002/mds.22370 PMC2683428 19185016

[7] Louis ED, Agnew A, Gillman A, Gerbin M, Viner AS. Estimating annual rate of decline: prospective, longitudinal data on arm tremor severity in two groups of essential tremor cases. J. Neurol. Neurosurg. Psychiatry. 2011; 82: 761–765. DOI: 10.1136/jnnp.2010.229740 21436230 PMC3696191

[8] Louis ED. Essential tremor. Handb. Clin. Neurol. 2023; 196: 389–401. DOI: 10.1016/B978-0-323-98817-9.00012-0 37620080

[9] Louis ED, Gerbin M, Galecki M. Essential tremor 10, 20, 30, 40: clinical snapshots of the disease by decade of duration. Eur. J. Neurol. 2013; 20: 949–954. DOI: 10.1111/ene.12123 23521518 PMC3653981

[10] Gutierrez J, Park J, Badejo O, Louis ED. Worse and Worse and Worse: Essential Tremor Patients’ Longitudinal Perspectives on Their Condition. Front. Neurol. 2016; 7: 175. DOI: 10.3389/fneur.2016.00175 27790185 PMC5061994

[11] Louis ED, Ford B, Lee H, Andrews H, Cameron G. Diagnostic criteria for essential tremor: a population perspective. Arch. Neurol. 1998; 55: 823–828. DOI: 10.1001/archneur.55.6.823 9626774

[12] Louis ED, et al. The Washington Heights-Inwood Genetic Study of Essential Tremor: methodologic issues in essential-tremor research. Neuroepidemiology. 1997; 16: 124–133. DOI: 10.1159/000109681 9159767

[13] Elble RJ, Ondo W. Tremor rating scales and laboratory tools for assessing tremor. J. Neurol. Sci. 2022; 435: 120202. DOI: 10.1016/j.jns.2022.120202 35220111

[14] Louis ED, Ford B, Bismuth B. Reliability between two observers using a protocol for diagnosing essential tremor. Mov. Disord. Off. J. Mov. Disord. Soc. 1998; 13: 287–293. DOI: 10.1002/mds.870130215 9539343

[15] Louis ED, et al. Validity of a performance-based test of function in essential tremor. Arch. Neurol. 1999; 56: 841–846. DOI: 10.1001/archneur.56.7.841 10404986

[16] Handforth A, Bordelon Y, Frucht SJ, Quesada A. A pilot efficacy and tolerability trial of memantine for essential tremor. Clin. Neuropharmacol. 2010; 33: 223–226. DOI: 10.1097/WNF.0b013e3181ebd109 20838216

[17] Pérez–Dueñas B, et al. Characterization of tremor in phenylketonuric patients. J. Neurol. 2005; 252: 1328–1334. DOI: 10.1007/s00415-005-0860-6 15995796

[18] Sulica L, Louis ED. Clinical characteristics of essential voice tremor: A study of 34 cases. The Laryngoscope. 2010; 120: 516–528. DOI: 10.1002/lary.20702 20066728

[19] Gatto EM, Roca MCU, Raina G, Micheli F. Low Doses of Topiramate are Effective in Essential Tremor: A Report of Three Cases. Clin. Neuropharmacol. 2003; 26: 294–296. DOI: 10.1097/00002826-200311000-00006 14646607

[20] Seijo-Martínez M, et al. Prevalence of Essential Tremor on Arosa Island, Spain: a Community-based, Door-to-Door Survey. Tremor Hyperkinetic Mov. N. Y. N. 2013; 3: tre-03–192–4299-1. DOI: 10.5334/tohm.130 PMC3779822 24116344

[21] Dogu O, et al. Prevalence of essential tremor: door-to-door neurologic exams in Mersin Province, Turkey. Neurology. 2003; 61: 1804–1806. DOI: 10.1212/01.WNL.0000099075.19951.8C 14694055

[22] Silahli NY, Turkdogan D. Prevalence of Potential Essential Tremor Cases in Turkish Adolescents According to The WHIGET Classification. Turk. Arch. Pediatr. 2022; 57: 323–328. DOI: 10.5152/TurkArchPediatr.2022.21218 35781236 PMC9131808

[23] Güler S, Caylan A, Turan FN, Dağdeviren N. The prevalence of essential tremor in Edirne and its counties accompanied comorbid conditions. Neurol. Res. 2019; 41: 847–856. DOI: 10.1080/01616412.2019.1628409 31238803

[24] Rüegge D, et al. Tremor analysis with wearable sensors correlates with outcome after thalamic deep brain stimulation. Clin. Park. Relat. Disord. 2020; 3: 100066. DOI: 10.1016/j.prdoa.2020.100066 34316646 PMC8298798

[25] Silek H, Dogan M. Voice Analysis in Patients with Essential Tremor. J. Voice Off. J. Voice Found. 2023; S0892–1997(23): 00144–3. DOI: 10.1016/j.jvoice.2023.04.019 37336699

[26] Heldman DA, et al. Essential tremor quantification during activities of daily living. Parkinsonism Relat. Disord. 2011; 17: 537–542. DOI: 10.1016/j.parkreldis.2011.04.017 21570891 PMC3137659

[27] Elble R, et al. Reliability of a new scale for essential tremor. Mov. Disord. Off. J. Mov. Disord. Soc. 2012; 27: 1567–1569. DOI: 10.1002/mds.25162 PMC4157921 23032792

[28] Deveney CM, et al. Transcranial focused ultrasound for the treatment of tremor: A preliminary case series. Brain Stimulat. 2023; 17: 35–38. DOI: 10.1016/j.brs.2023.12.007 38128826

[29] Hollý P, et al. Essential and dystonic head tremor: More similarities than differences. Parkinsonism Relat. Disord. 2023; 115: 105850. DOI: 10.1016/j.parkreldis.2023.105850 37708603

[30] van Veen R, et al. The effect of tremor on disability assessment in chronic inflammatory demyelinating polyradiculoneuropathy. J. Peripher. Nerv. Syst. JPNS. 2023; 28: 58–68. DOI: 10.1111/jns.12528 36571466

[31] Ondo WG, Pascual B, Tremor Research Group. Tremor Research Group Essential Tremor Rating Scale (TETRAS): Assessing Impact of Different Item Instructions and Procedures. Tremor Hyperkinetic Mov. N. Y. N. 2020; 10: 36. DOI: 10.5334/tohm.64 PMC7546108 33101762

[32] Berry DS, Cosentino S, Louis ED. A prospective cohort study of familial versus sporadic essential tremor cases: Do clinical features evolve differently across time? J. Neurol. Sci. 2023; 454: 120854. DOI: 10.1016/j.jns.2023.12085437924593

[33] Delgado N, Berry DS, Louis ED. Distribution of rest tremor in patients with Essential Tremor: Does it lateralize with simple kinetic, postural, or intention tremors? Parkinsonism Relat. Disord. 2022; 102: 36–41. DOI: 10.1016/j.parkreldis.2022.07.018 35933821

[34] Bhatia KP, et al. Consensus Statement on the classification of tremors. from the task force on tremor of the International Parkinson and Movement Disorder Society. Mov. Disord. Off. J. Mov. Disord. Soc. 2018; 33: 75–87. DOI: 10.1002/mds.27121 PMC6530552 29193359

[35] Cohen J. Weighted kappa: nominal scale agreement with provision for scaled disagreement or partial credit. Psychol. Bull. 1968; 70: 213–220. DOI: 10.1037/h0026256 19673146

[36] Landis JR, Koch GG. The measurement of observer agreement for categorical data. Biometrics. 1977; 33: 159–174. DOI: 10.2307/2529310 843571

[37] Stacy MA, et al. Assessment of interrater and intrarater reliability of the Fahn-Tolosa-Marin Tremor Rating Scale in essential tremor. Mov. Disord. Off. J. Mov. Disord. Soc. 2007; 22: 833–838. DOI: 10.1002/mds.21412 17343274

