# Supplementary material for: WHIGET and TETRAS Ratings of Action Tremor in Patients with Essential Tremor: Substantial Association and Agreement
Source: Tremor Other Hyperkinet Mov (N Y). 2024 Mar 27;14:14. doi: 10.5334/tohm.874 (PMC10976980; doi:10.5334/tohm.874)
Supplement: Supplemental Table 1. — Cross-Tabulations of WHIGET and TETRAS Ratings. [file tohm-14-1-874-s1.pdf]

Supplemental Table 1: Cross-Tabulations of WHIGET and TETRAS Ratings

Spiral, dominant

WHIGET RATING

	0.0	0.5	1.0	1.5	2.0	3.0	4.0
TETRAS RATING							
0	7	0	1	0	0	0	0
1	0	2	6	2	0	0	0
2	0	0	2	13	22	0	0
3	0	0	0	0	7	16	0
4	0	0	0	0	0	0	1

Spiral, non-dominant

WHIGET RATING

	0.0	0.5	1.0	1.5	2.0	3.0	4.0
TETRAS RATING							
0	3	0	1	0	0	0	0
1	0	2	5	2	0	0	0
2	0	0	7	17	19	0	0
3	0	0	0	0	5	14	0
4	0	0	0	0	0	2	2

Finger to nose, dominant

WHIGET RATING

	0.0	0.5	1.0	1.5	2.0	3.0	4.0
TETRAS RATING							
0.0	4	0	0	0	1	0	0
1.0	0	0	7	1	1	0	0
1.5	0	0	20	2	2	0	0
2.0	0	0	2	4	31	0	0
2.5	0	0	0	0	2	2	0
3.0	0	0	0	0	0	0	0
3.5	0	0	0	0	0	0	0
4.0	0	0	0	0	0	0	0

Finger to nose, non-dominant

WHIGET RATING

	0.0	0.5	1.0	1.5	2.0	3.0	4.0
TETRAS RATING							
0.0	3	0	1	0	1	0	0
1.0	0	0	3	0	0	0	0
1.5	0	0	12	3	1	0	0
2.0	0	1	1	4	45	0	0
2.5	0	0	0	0	2	1	0
3.0	0	0	0	0	0	0	0
3.5	0	0	0	0	0	1	0
4.0	0	0	0	0	0	0	0

Outstretched, dominant

WHIGET RATING

	0.0	0.5	1.0	1.5	2.0	3.0
TETRAS RATING						
0.0	18	0	0	0	0	0
1.0	0	5	7	0	0	0
1.5	0	0	10	0	0	0
2.0	0	0	1	9	22	0
2.5	0	0	0	0	0	3
3.0	0	0	0	0	0	3
3.5	0	0	0	0	0	1
4.0	0	0	0	0	0	0

Outstretched, non-dominant

WHIGET RATING

	0.0	0.5	1.0	1.5	2.0	3.0
TETRAS RATING						
0.0	11	0	0	0	0	0
1.0	0	7	2	1	0	0
1.5	0	0	18	1	0	0
2.0	0	0	2	13	17	0
2.5	0	0	0	0	3	1
3.0	0	0	0	0	0	2
3.5	0	0	0	0	0	0
4.0	0	0	0	0	0	1

Wingbeat, dominant

WHIGET RATING

	0.0	0.5	1.0	1.5	2.0	3.0
TETRAS RATING						
0.0	9	0	0	0	0	0
1.0	0	6	7	0	0	0
1.5	0	0	10	0	1	0
2.0	0	0	0	14	19	1
2.5	0	0	0	0	2	2
3.0	0	0	0	0	0	6
3.5	0	0	0	0	0	1
4.0	0	0	0	0	0	1

Wingbeat, non-dominant

WHIGET RATING

	0.0	0.5	1.0	1.5	2.0	3.0
TETRAS RATING						
0.0	7	0	0	0	0	0
1.0	0	7	6	1	0	0
1.5	0	0	15	1	0	0
2.0	0	0	1	9	24	0
2.5	0	0	0	0	0	2
3.0	0	0	0	0	0	3
3.5	0	0	0	0	0	1
4.0	0	0	0	0	0	1
